# Patient and Provider Recommendations for Improved Telemedicine User Experience in Primary Care: A Multi-Center Qualitative Study

**DOI:** 10.1089/tmr.2023.0002

**Published:** 2023-03-20

**Authors:** Saif Khairat, Prabal Chourasia, Kimberly A. Muellers, Katerina Andreadis, Jenny J. Lin, Jessica S. Ancker

**Affiliations:** ^1^ Carolina Health Informatics Program, University of North Carolina at Chapel Hill, Chapel Hill, North Carolina, USA.; ^2^ School of Nursing, University of North Carolina at Chapel Hill, North Carolina, USA.; ^3^ Sheps G. Cecil Center for Health Services Research, University of North Carolina at Chapel Hill, North Carolina, USA.; ^4^ Division of General Internal Medicine, Icahn School of Medicine at Mount Sinai, New York, New York, USA.; ^5^ Department of Psychology, Pace University, New York, New York, USA.; ^6^ Department of Population Health Sciences, Weill Cornell Medicine, New York, New York, USA.; ^7^ Department of Population Health, New York University Grossman School of Medicine, New York, New York, USA.; ^8^ Department of Biomedical Informatics, Vanderbilt University Medical Center, Nashville, Tennessee, USA.

**Keywords:** telemedicine, primary care, user experience, providers, patients

## Abstract

**Objective:**

The purpose of this study was to explore telemedicine use and obtain actionable recommendations to improve telemedicine user experience from a diverse group of patients and providers.

**Methods:**

We interviewed adult patients and primary care providers (PCPs) across three National Patient-Centered Clinical Research Network (PCORnet) sites in New York City, North Carolina, and Florida. Both patients and providers could participate via phone or videoconferencing; patients could complete the interview in English or Spanish. Spanish interviews were conducted by a member of the research team who spoke Spanish fluently. Interviews were audio-recorded, transcribed verbatim, and when necessary, professionally translated.

**Results:**

We interviewed 21 PCPs and 65 patients between March and October 2021. We found that patients' and providers' perspectives on ways to improve the telemedicine experience focused on three recommendation themes: (1) expectations of care provided via telemedicine, (2) innovations to support usability, and (3) alleviation of physician burden. Key recommendations were related to expectations regarding (1) care provided, for example, adding educational content for the patients, and clarity about long-term payment models; (2) support innovation to improve telemedicine usability, for example, providing patients with remote monitoring devices, integrating in-home testing and nursing evaluation; (3) and reduce physician burden, for example, virtual rooming, reimbursement of time spent outside of the telemedicine encounter.

**Discussion:**

Primary care patients and providers see merit in telemedicine. However, both groups recommended novel ways to improve the quality of care and user experience. Findings from this article suggest that policymakers would be best served by addressing current gaps in patient digital literacy by creating technical support strategies, and gaps in telemedicine reimbursement to present an equitable form of payment.

## Introduction

Telemedicine use has rapidly expanded, and it is now an integral part of health care delivery.^1^ It has proven to be instrumental in connecting physicians with patients during the COVID-19 pandemic.^1^ Benefits of telemedicine include increased access to care, reduced waiting and travel time, and providing more options and flexibility for patients.^2^ For providers, it can reduce crowding in waiting rooms and allow them to care for a wider patient population including those in remote areas. Although telemedicine has been useful in connecting physicians and patients, its sudden large-scale adoption in the face of the COVID-19 pandemic exposed challenges associated with its utilization, which can result in poor user experiences.

One barrier is the lack of telemedicine training and education for physicians, health care workers, and patients. A second important barrier involves inequitable access to broadband internet and devices to use it so that telemedicine may worsen health inequities.^3^ Both these barriers contribute to the user (both patient and provider) experience of telemedicine.

User experience is defined as “a person's perceptions and responses that result from the use or anticipated use of a product, system or service.”^4^ Positive user experience is critical for the adoption, acceptability, and effectiveness of telemedicine.^5,6^ User experience is closely associated with patient satisfaction, an important quality-of-care indicator. Multiple studies have identified important factors associated with improved patient satisfaction with telemedicine such as ease of use, low cost, better communication, and decreased travel time.^2,7–14^

Another study reported that while patients found telemedicine to be less stressful, associated challenges included time lag, video freezing, uncertainty in virtual waiting room, technology problems (some needing transfer to phone), and unclear expectations leading to poor patient satisfaction.^15,16^

In parallel, studies have shown that provider satisfaction with telemedicine is associated with having administrative support and reliable technology, being able to provide input in its development, ease of use, and adequate reimbursement.^17–21^ Provider satisfaction is closely linked to provider acceptance, which has been found to be the most important factor determining success of telemedicine.^22^ Providers have been less satisfied with telemedicine as compared with in-person visits due to perceived reduced doctor-patient communication.^23^ The perceived ease of use and usefulness of telemedicine services are dominant factors affecting provider satisfaction.^24^

Despite prior studies analyzing provider and patient user experiences with telemedicine, there is a critical gap around actionable recommendations from end-users to improve the user experience. Improving user experience for both providers and patients in various settings (urban, suburban, rural) and age groups is critical for wider telemedicine acceptance and success. The purpose of this study was to explore telemedicine use and obtain actionable recommendations to improve telemedicine user experience from a diverse group of patients and providers.

## Methods

### Participants

We identified adult patients and primary care providers (PCPs) across three National Patient-Centered Clinical Research Network (PCORnet) sites in New York City (urban), North Carolina (suburban), and Florida (rural). Using a definition adapted from the Medicare specialty designation, we defined adult primary care as practices in the fields of general practice, family practice, ambulatory internal medicine, preventive, and geriatric medicine.

Based on a sampling frame of 250 primary care practices and with the help of recruited clinician champions, we recruited participants (i.e., providers and patients) between March and October 2021 through several methods, including emails, patient registries, flyers, clinician referrals, and snowball referrals from participants. The clinician champions did not participate in the interviews.

Eligible patients were 18 years, English- or Spanish-speaking, able to participate via telephone or videoconferencing, and had at least one chronic disease diagnosis. Eligible PCPs worked in primary care at one of the recruitment sites. Maximum variation sampling^25^ was used to sample participants of different ages, races, ethnicities, geographic locations, and levels of technology experience.

To ensure diverse representation, the study team developed a screening checklist to ensure eligibility assessments were being conducted uniformly across sites and quotas to avoid over-sampling certain groups. The study protocol was approved by the Biomedical Research Alliance of New York institutional review board.

### Measures

Semi-structured interview guides were developed in collaboration with our stakeholder board, which included patients, providers, payers, and information technology experts. Research staff conducted individual interviews asking about participants' experiences with telemedicine during the pandemic. Both patients and providers could participate via phone or videoconferencing; patients could complete the interview in English or Spanish. Spanish interviews were conducted by a member of the research team who spoke Spanish fluently. Interviews were audio-recorded, transcribed verbatim, and when necessary, professionally translated.

### Analysis

Coders developed code keys for providers and patient transcripts based on *a priori* domains from the interview guides and emergent codes. Three researchers (K.A., K.A.M., J.J.L.) coded transcripts independently and met to compare codes and resolve discrepancies.

Stakeholder board did not aid in the coding or results interpretation. Data were analyzed using interpretive description,^26^ an approach previously applied to health care experiences.^27^ Iterative analysis was conducted in parallel with recruitment, and recruitment concluded when data saturation was achieved.^28^ Final codes were captured using Dedoose Version 9.0.46 (Los Angeles, CA).

For the current study, we retrieved all text with codes related to recommendations for improvement in telemedicine. Two domain expert researchers (S.K., P.C.) independently ranked the novelty of all codes on a 3-point scale (1 = low, 3 = high). Novelty was defined as innovative ideas, based on domain experts assessment, that can improve the telemedicine user experience. Then, the average scores were calculated and those codes with three were included in this analysis, which represented high novelty.

The mean was used instead of the median since there were no outliers in the ranking since only two domain experts ranked the codes. Those codes were then categorized into themes based on patient and provider-based reported outcomes from the Benson framework.^29^ The Benson framework is a comprehensive taxonomy of short generic measures covering both patient-reported and provider-reported outcomes, which enables the categorization of participant responses into mutually exclusive themes.

## Results

We interviewed 21 PCPs and 65 patients between March and October 2021. Of the patients, 60% were female and 42% self-identified as White, 25% as Black, 23% as Hispanic, 9% as other, and 1% as Asian. Half were between the ages of 41–65 years, 26% were <40 years, and 22% were >65 years. Two of the interviews were conducted in Spanish. Of the PCPs, 62% were female and 48% self- identified as White, 24% as Asian, 14% as Hispanic, 9% as Black, and 5% as other.

The majority were between 41 and 60 years, with 29% <40 years and 14%> 60 years (Table 1). Patients and PCPs were recruited uniformly from each of the three sites in New York, Florida, and North Carolina. Among the 21 PCPs, 7 were recruited from New York, 8 from Florida and 6 from North Carolina. Among patients,—were recruited from New York,—from Florida, and—from North Carolina. On average, patient interviews lasted 20–25 min, whereas provider interviews ranged from 30 to 40 min.

**Table 1.** Demographics of Participants

Characteristics	Patients (***n*** = 65)	Providers (***n*** = 21)
Age group, *n* (%)		
<25	2 (3)	—
25–40	17 (26)	6 (29)
41–60 (65)	32 (49)	12 (57)
>60 (65)	14 (21)	3 (14)
Female, *n* (%)	39 (60)	13 (62)
Race/Ethnicity, *n* (%)		
Black	16 (25)	2 (10)
White	27 (42)	10 (48)
Asian	1 (1)	5 (24)
Hispanic	15 (23)	3 (14)
Location, *n* (%)		
Florida	21 (32)	8 (38)
New York	24 (37)	7 (33)
North Carolina	20 (31)	6 (29)
Type of practice, *n* (%)		
Academic practice	—	1 (0.5)
FQHC/community	—	4 (19)
Teaching/training practice	—	16 (76)

Only patient participants were asked about their primary language, and only provider participants were asked about the type of practice they are working in. Patient participants were asked about their primary language and whether they preferred to take the interview in English, or Spanish. Four participants with a “Spanish” primary language selected to do the interview in English, while two selected to do the interview in Spanish. Two participants selected “Other” as their primary language but felt comfortable conducting the interview in English.

FQHC, Federally Qualified Health Centers.

We found that patients' and providers' perspectives on ways to improve the telemedicine experience focused on three recommendation themes: (1) expectations of care provided via telemedicine, (2) innovations to support usability, and (3) alleviation of physician burden (Table 2, Table 3). Four patients and five providers contributed to theme 1, six patients and five providers contributed to theme 2, and six providers contributed to theme 3.

**Table 2.** Recommendations from Patients to Improve the User Experience Categorized by Themes: (1) Care Provided, (2) Innovation, and (3) Individual Care

Benson framework	Quote	Patient
Care provided	Where combining the video conferencing technology with some of those in-home lab options, where like, they could—**for patients who have limited mobility or disabilities so they could send like blood**—what do you have to do, blood work. If they could offer those options, where they can send that stuff directly to patients' home and do those results and then send it off and then, you know, get the result and send it back to them and be able to (inaudible) that way (P1)	25–40 year-old English North Carolina
Care provided	I think the results that are uploaded to these portals for the lab results, it would be very helpful if there was a **narrative interpretation by someone.** (P2)	41–65 year-old English North Carolina
Care provided	Like I think it would be better if there was a **nurse to check your vitals, check your lungs at home before your appointment** or wherever you are. Just things like that, you know. But I don't know if insurance will ever cover things like that you know. (P3)	19–24 year-old English North Carolina
Innovation	the only thing that I could say is to get a little more check—for example, I was very impressed when **Dr. Sue could show me some screenshots of lab results** or the cancer process or the surgery process, and so that was very helpful, I think, again, when I'm talking to the other physicians, perhaps they could put the screenshot in my lab results, or whatever it is that they were talking about, so just having them a little more checked out, and the ability to do that, I think would be helpful. (P4)	>65 year-old English Florida
Innovation	I think something that could be more implemented would be just using—**sharing screens more often**, just using more of the features of Zoom. So, if somebody needs a pamphlet for something, they would just have those links accessible, because right now, as far as I know, they don't—or at least my asthma/allergy specialist just had like the paper pamphlets in the office. Or just more detailed information like readily at hand like in a link they could send in a chat kind of thing. (P5)	41–65 year-old English Florida
Innovation	Especially during the summer. I travel a lot––to Europe, to Asia. Sometimes you really want to reach out to your family, your, your family doctor. You know, I think you could actually do this virtual telemedicine until we say, you know, **maybe your doctor said hey, you need to go to a local doctor, you need to get some blood tests,** and so on and so forth. I think that actually, you know, could be quite interesting to have that option. (P6)	41–65 year-old English Florida
Innovation	It would be nice if they would allow—**like if there are group meetings.** The option of, say if your child was sick, but you still wanted to participate and couldn't be there, that they allow in-person and telehealth. Like somebody on the screen to be able to participate. (P7)	25–40 year-old English North Carolina
Individual care	“I've noticed the ones that have it set up where you get a kit at home is easier because they can read the temperature off the screen and you attach that thing right here and they can read your heart rate.” “But if that **was made affordable to where people who choose telehealth can have that kit**, then I think that would be good across the board.” (P8)	25–40 year-old English North Carolina
Individual care	I'm just thinking **in terms of domestic violence situations**, if the provider said turn your volume down; I'm going to ask you something. Or something along those lines. Be like are you in a safe place and you can nod your head or shake your head. Yeah, something along those lines just to kind of gauge, also so they know that maybe there's a work going on that you're not going to say. I think that telemedicine can be powerful in that way because you never know what's happening right outside the screen. (P9)	25–40 year-old English North Carolina
Individual care	I've never had **provider-utilized chat functions or doing any chatting on virtual visits**, but I think that would also be a really strong way to just gauge people's safety situations. (P10)	25–40 year-old English North Carolina

**Table 3.** Recommendations from Medical Providers to Improve the User Experience Categorized by themes: (1) Care Provided, (2) Innovation, and (3) Individual Care

Benson framework	Quote	Primary care provider
Care provided	From the world's perspective, if it was, if everybody had the same access, like all the insurance covered it, then that'll be good because sometimes we end up making a visit and then the patient gets a big bill. (P11)	FQHC/community Florida
Care provided	I mean, as a patient, like if I go to MyChart, I don't see like a link with its tutorial. Like a YouTube video, if you're going to make it, this is how it works. I mean that would be something really simple to have, to have patients while they wait. (P12)	FQHC/community Florida
Care provided	But if they don't have MyChart, they won't get AVS. So, in that regard, I always say, can you have pen and paper ready? Because this is my main thing that I want you to do, number one, number two, number three. At least three we can retain, especially for my elder population of patients. (P13)	FQHC/community New York
Care provided	If you call someone to talk to them about test results and spend 10, 15, 20 minutes on the phone, those are all billable visits. But if you don't have that as an expectation that that's, what's going to happen, then patients are not going to be particularly happy with that. (P14)	Teaching/training practice North Carolina
Care provided	We don't know how long we're going to be able to be reimbursed at the same rate, people are kind of unwilling to invest in that right now. (P15)	Teaching/training practice Florida
Innovation	So the virtual rooming could be done by a robot. So if a robot or whoever, a person, could do that and get everything all set up and—yeah, they could even check their medications or put in the chief complaint. (P16)	Teaching/training practice Florida
Innovation	If a physician is getting on and spending their first ten minutes of the visit doing tech support, that's very expensive and not probably the best model. (P17)	Teaching/training practice North Carolina
Innovation	Yes, I think as much as it can be integrated into the EHR and again one click. (P18)	Teaching/training practice North Carolina
Innovation	I think the more that we have care pathways like that that also integrate teams that can add some of these really important things without having to think of it every time, like oh, we haven't done inhaler teaching. (P19)	Teaching/training practice North Carolina
Innovation	For our patients with chronic disease, to be able to give them all devices where they could—the tools to track their chronic conditions at home. (P20)	Teaching/training practice New York
Innovation	Our care assistants do call the patients in advance for telemedicine. So, they're supposed to do essentially what they do in an office visit, go through medications and check on some components of health maintenance and offer support. But I don't think that they're as successful in offering support for how to use the interface. So maybe a little bit of education on how to use the interface. (P21)	Teaching/training practice North Carolina
Innovation	“Not only would it be easier for them, if there was some sort of universal physical exam technology that everybody could get.” “But if Sinai said you sign up for my chart, and we give you this kit of easy-to-use devices, and we put them here, and you use your smart phone through it, that would be helpful”. “But that would be helpful for the patient, I think it would be more up to date, I think the doctor would be more comfortable.” (P22)	Academic practice New York
Individual care	A way to track time spent that doesn't necessarily build a patient but still gives the provider credit for their time. ‘Cause I think we spend a lot of time doing things we don't get credit for. (P23)	Teaching/training practice North Carolina
Individual care	Having more breaks built in in-between visits also. So that, not only when you can take time to—you never know what happens—you could talk to a patient in one visit during which their loved ones just passed away and it's emotionally charged visit and then you're supposed to—you know, as Epic was telling me in notifications, other one's ready for you in 10 minutes. (P24)	Teaching/training practice New York
Individual care	Physical exam—that kind of goes to that whole thing, physical exam technology. I don't think that there's a significant comfort among doctors for physical exam technology. (P25)	Academic practice New York
Individual care	“I think from the provider perspective, I would love like getting, you know, being able to rework my clinic in a way that maybe I have to see less patients, but I have like time set aside for like tele-health billed phone calls to talk over test results and other things.” “But if I'm on the phone with you for 20 minutes doing coordination of care that counts for nothing.” (P26)	Teaching/training practice North Carolina

AVS, after visit summary; EHR, electronic health records.

### Theme 1: recommendations around the expectations for the care provided via telemedicine (Benson framework: care provided)

Four patients and five providers contributed to this theme. One provider recommended educational videos, such as “YouTube videos” (P12) for patients to improve their understanding of the telemedicine visit, especially while waiting for the telemedicine visit to begin. In addition, patients, especially older adults, may be unable to remember all the information discussed with the provider during the telemedicine visit, and with telemedicine visits, they do not receive a printed after-visit summary. One provider offered a solution to mitigate this by reminding patients to write important instructions: “can you have pen and paper ready? Because this is my main thing that I want you to do, number one, number two, number three” (P13).

The PCPs also emphasized the importance of managing patient expectations regarding insurance coverage and billable visits since telemedicine visits may not always be covered by insurance. As providers stated, the “patient gets a big bill” (P11) and “then patients are not going to be particularly happy with that” (P14). Uncertainty in telemedicine reimbursement can also hinder long-term investment to build and support a robust telemedicine infrastructure; as one provider noted, “we don't know how long we're going to be able to be reimbursed at the same rate, people are kind of unwilling to invest in that right now” (P15).

Clarity about long-term payment models for telemedicine visits from the government and private insurance companies will ensure long-term sustainability and development of innovative telemedicine platforms and improve patient care and health care access.

Telemedicine can be a means to assess home safety situations for patients. Providers voiced that educating PCPs of best-practices and integrating more telemedicine functions, beyond audio/video functions, can expand the unique opportunities offered by telemedicine visits compared with routine clinic visits. Patients offered several recommendations to support PCPs: “if the provider said turn your volume down; I'm going to ask you something—are you in a safe place and you can nod your head or shake your head” (P9).

Another patient suggested using “chat function [to] gauge people's safety situations” (P10). Patients also expressed interest in staying connected with their PCP via “virtual telemedicine” (P6) while travelling and “group meetings” (P7) with the ability for other family members to join remotely.

### Theme 2: recommendations to support innovations to improve telemedicine usability (Benson framework: innovation)

Five providers and six patients contributed to this theme. Several providers recommended that patients be provided with devices (“some sort of universal physical exam technology that everybody could get” or a “kit of easy-to-use devices” [P22]) to facilitate remote monitoring of their chronic conditions. These could be “the tools to track their chronic conditions at home” (P20). Similarly, one patient recommended an affordable vital monitoring kit that “can read the temperature off the screen [and] read your heart rate” (P8) to improve telemedicine visit care. Providing these tools to patients will also enable providers to conduct more comprehensive investigation; as a provider mentioned, with such tools, the “doctor would be more comfortable” (P22).

Patients also expressed that integrating in-home testing and nursing evaluation into the telemedicine visit would improve the telemedicine experience, especially for patients with “limited mobility or disabilities” (P1). Patients also saw benefits with an in-home visit by a “nurse to check your vitals, check your lungs at home before your appointment” (P3). One patient recommended the addition of a “narrative interpretation” (P2) of test results to make them easier for patients to understand.

During telemedicine visits, ancillary staff may not be available for pre-visit medication reconciliation. This causes providers to spend valuable and limited telemedicine visit time reviewing patients' home medications. One provider suggested using an automated system for virtual rooming to “get everything all set up.… they could even check their medications or put in the chief complaint” (P16).

Another important recommendation from providers is integrating telemedicine interfaces with institutional electronic health records (EHRs) to make the interface more user-friendly and reduce click burden. One provider recommended, “as much as [telemedicine] can be integrated into the EHR and again one click” (P18). There is also a need for innovation in designing and creating intuitive “care pathways” (P19) built into visits that can also serve as a visual reminder of “important things without having to think of it every time, like oh, we haven't done inhaler teaching” (P19).

On the other hand, patients recommended telemedicine functionality that would allow for “sharing screens more often” (P5), such as lab results (“perhaps they could put the screenshot in my lab results” [P4]) and links for information provided to patients during a visit: “So, if somebody needs a pamphlet for something, they will just have those links accessible” (P5).

### Theme 3: recommendations to alleviate physician burden with telemedicine (individual care)

Six providers contributed to this theme. Technology issues can negatively impact the telemedicine user experience and make it more expensive if providers need to provide “tech support” (P17) during the visit. Similarly, inadequately trained care assistants may not be “successful in offering support for how to use the interface” (P21) to the patients. Improving technical training for care assistants for simple issues and adequate technical support for complex issues can improve telemedicine visits and care.

Although physical examination is an important part of clinical evaluation, the ability to perform detailed physical examinations is limited in telemedicine. The current technologies used for physical examination are inadequate. This adds to the physician's burden by spending more time on subjective and observational assessments to compensate for physical examination limitations.

One provider stated, “I don't think that there's a significant comfort amongst doctors for physical exam technology” (P25). Effective integration of state-of-the-art remote examination technologies can improve providers' trust in and satisfaction with doing telemedicine well.

The PCPs do not always get credit for time spent on important patient care activities like reviewing labs, imaging, and updating patients. One provider noted that the ability to set aside time for things like “telehealth billed phone calls to talk over test results” (P26) can allow providers to see fewer patients. Building a system that “gives the provider credit for their time” (P23) spent taking care of the patient outside the clinical encounter can facilitate better focus on quality (over quantity) and can significantly reduce provider burnout.

Back-to-back telemedicine visits do not allow PCPs additional time to address patients' complaints. Also, intermittent notifications about the next visit during an “emotionally charged visit” (P24) can distract providers from addressing patients' concerns. One provider suggested that having “more breaks built in between visits” (P24) can help address this issue.

## Discussion

This multi-site study investigated the perceptions of patients and providers regarding ways to improve the user experience during telemedicine encounters. We report actionable recommendations from end-users based on their experiences with telemedicine visits during the COVID-19 pandemic. Recommendations were categorized into three main themes (1) expectations for the care provided via telemedicine, (2) innovations to improve telemedicine usability, and (3) alleviation of physician burden in telemedicine.

To enhance the quality of care provided, patients suggested rethinking the current telemedicine visit protocol to include domestic violence situations. Recommendations for providers to always ask the patient if they are in a safe place and then, to allow patients to communicate back in verbal or non-verbal cues (head shake, type in chat, etc.) were introduced. Another recommendation was to provide at-home lab options to enable patients to send their results to their provider before their telemedicine visit.

At-home kits can help providers better understand the patient's condition and hence, improve their decision-making abilities in the virtual space. The ability for patients to access provider notes and after-visit summary can improve adherence to the care plan.

Patients reported that more innovation is still needed to improve telemedicine usability. Patients stated that the ability to screen share during a telemedicine visit can empower patients by allowing providers to share educational resources to help with self-management or lab results to educate patients about their health status. Providers also suggested that integrating the telemedicine platform into the EHR can improve workflow automation and reduce documentation burden.

With regards to recommendations for reducing physician burden when using telemedicine, providers suggested integrating breaks in between telemedicine visits to allow providers to recalibrate especially after telemedicine visits where unpleasant news were discussed. Also, providers recommended reimagining how systems could better assess providers' time spent in patient care for reimbursement purposes. For instance, time spent on the phone for team coordination or resolving technical issues are not billable although they are clinically relevant.

To ensure meaningful implementation of telemedicine, decision makers and policy makers are encouraged to reconsider how providers' time is accounted for in the reimbursement cycle. It is not enough to bill for only the time spent in the telemedicine encounter without accounting for the supplemental tasks pre- and post-visit.

Another recommendation to reduce physician burden was to provide telemedicine usability support such as having ancillary staff call patients before their telemedicine visit to review medications and obtain necessary information. However, care assistants are often not trained to provide technical assistance to patients with limited digital literacy. Several providers indicated that lack of usability support for patients hinders the telemedicine experience and has cost implications due to prolonged visit duration. More ancillary support will allow physicians to utilize their valuable time for clinical evaluation, counseling, and patient support.

Previous studies that offered recommendations on telemedicine best-practices were based on expert opinions and mainly focused on implementation, policy, and visit etiquette.^30–32^ However, they were lacking in direct recommendations from end-users on the optimization of the telemedicine user experience. We report specific recommendations to improve the telemedicine user experience by improving the quality of care provided (i.e., adding educational content for the patients, managing patients' expectations regarding insurance coverage, clarity about long-term payment models, establishment of protocols to assess domestic violence); supporting innovation to improve telemedicine usability (i.e., providing patients with remote monitoring devices, integrating in-home testing and nursing evaluation); and to reduce physician burden (i.e., virtual rooming, more ancillary as well as technical support, reimbursement of time spent outside of the telemedicine encounter).

This study has several limitations. Although we worked systematically to identify and recruit diverse patient and provider participants, our patients' perspectives may reflect those of individuals more engaged with the health system. In addition, despite our efforts to recruit Spanish-speaking patient participants, we were only able to conduct two interviews in Spanish and did not include other languages as an option. Thus, we cannot describe the experiences of other patients whose communication might be even more affected in virtual settings.

Though our study findings are based on qualitative interviews and might not be generalizable to a population beyond primary care, they provide meaningful insights into patients' and providers' experience and suggestions to improve telemedicine. Of the three sites, only one site recruited telemedicine champions and no information was obtained about the number of sites they represented. It is plausible that recruitment through champions may introduce bias in the sample at that site.

Similarly for the other two sites, no data were obtained regarding how many practices were represented. For each provider, we recorded the practice type but not the exact practice that the providers came from as it was not responsive to the research question.

## Conclusion

In summary, primary care patients and providers see merit in telemedicine. However, both groups recommended novel ways to improve the quality of care and user experience. Key recommendations were related to expectations regarding (1) care provided, for example, adding educational content for the patients, and clarity about long-term payment models; (2) support innovation to improve telemedicine usability, for example, providing patients with remote monitoring devices, integrating in-home testing and nursing evaluation; (3) and reduce physician burden, for example, virtual rooming, reimbursement of time spent outside of the telemedicine encounter.

Findings from this article suggest that policymakers would be best served by addressing current gaps in patient digital literacy by creating technical support strategies, and gaps in telemedicine reimbursement to present an equitable form of payment to providers.

## Abbreviations Used

AVS: after visit summary
EHR: electronic health records
FQHC: Federally Qualified Health Centers
PCPs: primary care providers
PCORnet: Patient-Centered Clinical Research Network

## References

[1] Monaghesh E, Hajizadeh A. The role of telehealth during COVID-19 outbreak: A systematic review based on current evidence. BMC Public Health 2020;20(1):1193; doi: 10.1186/s12889-020-09301-4 32738884 PMC7395209

[2] Kruse CS, Krowski N, Rodriguez B, et al. Telehealth and patient satisfaction: A systematic review and narrative analysis. BMJ Open 2017;7(8):e016242; doi: 10.1136/bmjopen-2017-016242 PMC5629741 28775188

[3] Iacobucci G. Online consulting enthusiasts must engage with criticism, says GP leader. BMJ 2018;362(k4045; doi: 10.1136/bmj.k4045 30254036

[4] Mirnig A, Meschtscherjakov A, Wurhofer D, et al. A formal analysis of the ISO 9241-210 definition of user experience. 2015. https://dl.acm.org/doi/10.1145/2702613.2732511 [Last accessed: March 9, 2023].

[5] Khairat S, Lin X, Liu S, et al. Evaluation of patient experience during virtual and in-person urgent care visits: Time and cost analysis. J Patient Exp 2021;8(2374373520981487; doi: 10.1177/2374373520981487 PMC8205332 34189260

[6] Khairat S, Bohlmann A, Wallace E, et al. Implementation and evaluation of a telemedicine program for specialty care in North Carolina correctional facilities. JAMA Network Open 2021;4(8):e2121102; doi: 10.1001/jamanetworkopen.2021.21102 34398207 PMC8369360

[7] Iqbal A, Raza A, Huang E, et al. Cost effectiveness of a novel attempt to reduce readmission after ileostomy creation. JSLS 2017;21(1); doi: 10.4293/jsls.2016.00082 PMC5266511 28144122

[8] Dias AE, Limongi JCP, Hsing WT, et al. Telerehabilitation in Parkinson's disease: Influence of cognitive status. Dement Neuropsychol 2016;10(4):327–332; doi: 10.1590/s1980-5764-2016dn1004012 29213477 PMC5619273

[9] Hoaas H, Andreassen HK, Lien LA, et al. Adherence and factors affecting satisfaction in long-term telerehabilitation for patients with chronic obstructive pulmonary disease: A mixed methods study. BMC Med Inform Decis Mak 2016;16:26; doi: 10.1186/s12911-016-0264-9 26911326 PMC4766676

[10] Jacobs J, Ekkelboom R, Jacobs J, et al. Patient satisfaction with a teleradiology service in general practice. BMC Fam Pract 2016;17:17; doi: 10.1186/s12875-016-0418-y 26864118 PMC4748486

[11] Georgsson M, Staggers N. Quantifying usability: An evaluation of a diabetes mHealth system on effectiveness, efficiency, and satisfaction metrics with associated user characteristics. J Am Med Inform Assoc 2016;23(1):5–11; doi: 10.1093/jamia/ocv099 26377990 PMC4713903

[12] Polinski JM, Barker T, Gagliano N, et al. Patients' satisfaction with and preference for telehealth visits. J Gen Intern Med 2016;31(3):269–275; doi: 10.1007/s11606-015-3489-x 26269131 PMC4762824

[13] Levy N, Moynihan V, Nilo A, et al. Addendum to: The mobile insulin titration intervention (MITI) for insulin glargine titration in an urban, low-income population: Randomized controlled trial protocol. JMIR Res Protoc 2015;4(4):e138; doi: 10.2196/resprot.5403 30578192 PMC6318147

[14] Müller KI, Alstadhaug KB, Bekkelund SI. Acceptability, feasibility, and cost of telemedicine for nonacute headaches: A randomized study comparing video and traditional consultations. J Med Internet Res 2016;18(5):e140; doi: 10.2196/jmir.5221 27241876 PMC4906238

[15] Gabrielsson-Järhult F, Kjellström S, Josefsson KA. Telemedicine consultations with physicians in Swedish primary care: A mixed methods study of users' experiences and care patterns. Scand J Prim Health Care 2021;39(2):204–213; doi: 10.1080/02813432.2021.1913904 33974502 PMC8293950

[16] Donaghy E, Atherton H, Hammersley V, et al. Acceptability, benefits, and challenges of video consulting: A qualitative study in primary care. Br J Gen Pract 2019;69(686):e586–e594; doi: 10.3399/bjgp19X704141 31160368 PMC6617540

[17] Nguyen M, Waller M, Pandya A, et al. A review of patient and provider satisfaction with telemedicine. Curr Allergy Asthma Rep 2020;20(11):72; doi: 10.1007/s11882-020-00969-7 32959158 PMC7505720

[18] Ariens LF, Schussler-Raymakers FM, Frima C, et al. Barriers and facilitators to ehealth use in daily practice: Perspectives of patients and professionals in dermatology. J Med Internet Res 2017;19(9):e300; doi: 10.2196/jmir.7512 28874336 PMC5605757

[19] Rho MJ, Choi IY, Lee J. Predictive factors of telemedicine service acceptance and behavioral intention of physicians. Int J Med Inform 2014;83(8):559–571; doi: 10.1016/j.ijmedinf.2014.05.005 24961820

[20] Huang JC. Innovative health care delivery system—A questionnaire survey to evaluate the influence of behavioral factors on individuals' acceptance of telecare. Comput Biol Med 2013;43(4):281–286; doi: 10.1016/j.compbiomed.2012.12.011 23375377

[21] Demiris G. Examining health care providers' participation in telemedicine system design and implementation. In: AMIA Annual Symposium proceedings/AMIA Symposium AMIA Symposium 2006;2006, 906. PMC1839385 17238525

[22] Wade VA, Eliott JA, Hiller JE. Clinician acceptance is the key factor for sustainable telehealth services. Qual Health Res 2014;24(5):682–694; doi: 10.1177/1049732314528809 24685708

[23] Liu X, Sawada Y, Takizawa T, et al. Doctor-patient communication: A comparison between telemedicine consultation and face-to-face consultation. Intern Med 2007;46(5):227–232; doi: 10.2169/internalmedicine.46.1813 17329917

[24] Kissi J, Dai B, Dogbe CS, et al. Predictive factors of physicians' satisfaction with telemedicine services acceptance. Health Informatics J 2020;26(3):1866–1880; doi: 10.1177/1460458219892162 31854222

[25] Coyne IT. Sampling in qualitative research. Purposeful and theoretical sampling; merging or clear boundaries? J Adv Nurs 1997;26(3):623–630; doi: 10.1046/j.1365-2648.1997.t01-25-00999.x 9378886

[26] Hunt MR. Strengths and challenges in the use of interpretive description: Reflections arising from a study of the moral experience of health professionals in humanitarian work. Qual Health Res 2009;19(9):1284–1292; doi: 10.1177/1049732309344612 19690208

[27] Thorne S, Con A, McGuinness L, et al. Health care communication issues in multiple sclerosis: An interpretive description. Qual Health Res 2004;14(1):5–22; doi: 10.1177/1049732303259618 14725173

[28] Guest G, Namey E, Chen M. A simple method to assess and report thematic saturation in qualitative research. PLoS One 2020;15(5):e0232076; doi: 10.1371/journal.pone.0232076 32369511 PMC7200005

[29] Benson T. Measure what we want: A taxonomy of short generic person-reported outcome and experience measures (PROMs and PREMs). BMJ Open Qual 2020;9(1):e000789; doi: 10.1136/bmjoq-2019-000789 PMC7103852 32198234

[30] Daniel H, Sulmasy LS. Policy recommendations to guide the use of telemedicine in primary care settings: An American College of Physicians position paper. Ann Intern Med 2015;163(10):787–789; doi: 10.7326/m15-0498 26344925

[31] Khairat S, Pillai M, Edson B, et al. Evaluating the telehealth experience of patients with COVID-19 symptoms: Recommendations on best practices. J Patient Exp 2020;2374373520952975; doi: 10.1177/2374373520952975 PMC7705823 33294596

[32] Omboni S, McManus RJ, Bosworth HB, et al. Evidence and recommendations on the use of telemedicine for the management of arterial hypertension. Hypertension 2020;76(5):1368–1383; doi: 10.1161/HYPERTENSIONAHA.120.15873 32921195

